# The Role of School Environment in Physical Activity among Brazilian Adolescents

**DOI:** 10.1371/journal.pone.0131342

**Published:** 2015-06-22

**Authors:** Leandro Fórnias Machado de Rezende, Catarina Machado Azeredo, Kelly Samara Silva, Rafael Moreira Claro, Ivan França-Junior, Maria Fernanda Tourinho Peres, Olinda do Carmo Luiz, Renata Bertazzi Levy, José Eluf-Neto

**Affiliations:** 1 Faculdade de Medicina da Universidade de São Paulo, Departamento de Medicina Preventiva, São Paulo, Brasil; 2 Universidade Federal de Uberlândia, Faculdade de Medicina, Minas Gerais, Brazil; 3 Universidade Federal de Santa Catarina, Centro de Desportos, Santa Catarina, Brazil; 4 Universidade Federal de Minas Gerais, Departamento de Nutrição, Minas Gerais, Brazil; 5 Faculdade de Saúde Pública da Universidade de São Paulo, Departamento de Saúde Materno-Infantil, São Paulo, Brazil; Leibniz Institute for Prevention Research and Epidemiology (BIPS), GERMANY

## Abstract

**Objective:**

To analyze the association of physical activity facilities and extracurricular sports activities in schools with physical activity among adolescents.

**Methodology/Principal Findings:**

We used data collected for the National Survey of School Health in 2012. The national representative sample comprised 109,104 Brazilian students from 2,842 schools. We calculated the prevalence of participation in physical education classes, leisure-time physical activity, and total physical activity level. We also evaluated the following physical activity facilities: sports courts, running/athletics tracks, schoolyard with teacher-directed physical activities, swimming pools, locker rooms; and the offer of extracurricular sports activities. Schools with at least one physical activity facility had increased odds of participation in physical education (OR 1.59; 95% CI 1.20 to 2.10). However, in order to increase leisure-time physical activity (OR1.14; 95% CI 1.03 to 1.26) and total physical activity level (OR 1.15; 95% CI 1.06 to 1.24) at least four and two facilities, respectively, were necessary. Extracurricular sports activities in schools were positively associated with leisure-time physical activity and physical activity level. The number of sports courts and swimming pool in a school were associated with participation in physical education classes. Availability of sports courts, running/athletics tracks, and swimming pool in schools were associated with leisure-time physical activity. Total physical activity was associated with schools with sports courts, schoolyard with teacher-directed physical activities, and swimming pool.

**Conclusions:**

School-level characteristics have important potential to increase the possibility of engagement in physical activity in and out of school, and therefore have a fundamental role in promoting these practices.

## Introduction

Physical activity (PA) during adolescence is associated with health benefits on adiposity level, cardiometabolic health, mental health, academic self-concept, bone strength, and physical fitness[1, 2]. Notwithstanding the well-recognized PA benefits, in Brazil only 29% of adolescents meet the recommended PA guidelines (≥60 minutes of moderate-to-vigorous physical activity (MVPA) per day)[3], a scenario similar to other Latin American countries (10–20%) and worldwide (20%)[4].

From an ecological perspective, PA during adolescence is determined not exclusively by individual characteristics and choices but also by sociocultural and environmental factors (context)[5–7].Every day, choices about PA and opportunities to be physically active during school-time, and during recreational activities, are influenced by contextual characteristics (features of the school environment, safety, financial capacity, political and sociogeographical factors) [5–9].

Bearing in mind the amount of time spent in school, studies have shown that school environment explains an important part of the variance of PA in schoolchildren[10–12].Furthermore, non-availability of, or non-accessibility to, activities at school has been reported as important barriers to PA participation among adolescents [13]. Therefore, PA facilities in schools and the offer of extracurricular activities are important factors to be considered in the promotion of PA among adolescents.

Despite the importance of contextual characteristics, in low- and middle-income countries, demographic and biological characteristics are the most frequently investigated correlates, while the influence of environmental factors remains poorly comprehended, especially in relation to children and adolescents[14]. Indeed, in high-income countries, studies that investigated the school-context influence on PA participation have focused only on total physical activity level (PAL)[10–12].

In this study, we analyzed the role of availability of PA facilities (sports courts, running/athletics track, swimming pool, schoolyard with teacher-directed PA, locker rooms) and the offer of extracurricular sports activities in schools on physical education classes (PE classes), leisure-time physical activity (LTPA), and PAL among Brazilian adolescents. Finally, we investigated the variance of PE classes, LTPA and PAL by school-level.

## Material and Methods

### Study population, sampling and data collection

We used data collected for PeNSE (*Pesquisa Nacional de Saúde do Escolar*—National Survey of School Health), the main source of information on risk and protective factors for health in adolescents from public and private schools in Brazil[15].

PeNSE 2012 included a representative sample of students from the final (9^th^) year of elementary education, selected on the basis of a complex multi-stage, stratified, clustered probability design, conducted between April and September 2012[15]. The 2012 edition of PeNSE included, for the first time, contextual characteristics of schools in the survey. Briefly, the cluster sampling was performed in two stages in capital cities (with schools as primary units and classes as secondary units) and three stages in other municipalities (with municipalities as primary units, schools as secondary units and classes as tertiary units). From 3,004 eligible schools, a sample of 2,842 schools, with 110,873students (84% of the total number of students attending school), was enrolled in the survey. Of these students, 1,651 refused to participate, and 118 did not provide information on their sex and/or age (final response rate of 82.7%). The present study used data from 109,104 students at 2,842 schools. The sampling procedures and methods used by PeNSE are described in detail elsewhere[15].

The data were collected via an electronic, structured, self-administered questionnaire (using smartphones) based on the *Global School-Based Student Health Survey*[16], the *Youth Risk Behavior Surveillance System*[17], and other Brazilian research projects[18–21]. The questionnaire was composed of two main sections. The first, which concerned contextual characteristics of the school, was answered by the school director/coordinator. This section comprised 28 questions covering the following topics: monthly tuition and fee, number of enrolled students, school grades offered by the school, infrastructure (library, informatics room, canteen, sports courts, running/athletics track, schoolyard with teacher-directed PA, swimming pool, locker rooms), perception of violence in the surrounding area, council meetings, extracurricular sports activities and programs against tobacco use. The second section was self-administered by the students and covered individual characteristics comprising 12 modules: socio-demographic variables, nutrition, body image, physical activity, smoking, use of alcohol and other drugs, oral health, sexual behavior, violence, accidents, safety, and self-reported anthropometric measurements.

### Individual-level Measures

#### Measurement of physical activity

PA was assessed by means of a self-report questionnaire, which asked about frequency and duration of PA practice in the following domains: PE classes, LTPA, and active commuting to or from school. Regarding PE classes, the students were asked about frequency and duration during the preceding week. LTPA was related to frequency and duration in activities such as sports, dance, gymnastics, strength training, and combat. For active commuting to or from school, the frequency and duration of walking and bicycle use was considered.

We considered participation in each domain of physical activity based on weekly frequency. For PE classes, we considered ≥2 days/week (based on the number of classes offered per week in most Brazilian schools)[22, 23] and for LTPA≥1 day/week (to characterize participation or not). Despite some school environment variables (e.g. traffic safety around school, bicycle shed)[24] have been found to be associated with active commuting to or from school, PeNSE did not collect data on such characteristics. Variables collected for the PeNSE 2012 are potentially related only to practices inside the school (sports courts, running/athletics track, schoolyard with teacher-directed PA, swimming pool, locker rooms), for this reason, we did not analyze active commuting separately.

Additionally, the PAL was calculated by the sum of the weekly time spent engaged in PA, taking into account the frequency (0–7 days/week) and duration (e.g. ≤10, 10–19, 20–29, 30–39, 40–49, 50–59, ≥60 min/day), in the last seven days, in each domain (LTPA, PE classes, active commuting to and from school). Thus, PAL was categorized as <420 min/week or as ≥420 min/week, based on the level recommended by the World Health Organization (at least 60 minutes of MVPA daily)[25].

#### Sociodemographic characteristics

These included sex, age group (<13, 14–15, or >16 years); geographic region (north, northeast, southeast, south, or midwest), municipality (state capital or not), and mother’s education level (incomplete elementary education, complete elementary education, complete secondary education, or complete higher education). Due to the significant proportion of missing values for the mother’s education level (17%, n = 18,527), we performed multiple imputation using the chained equation technique. This method uses the distribution of the observed data to estimate a set of plausible values for the missing data. Thus, multiple imputation produces asymptotically unbiased estimates and standard errors and is asymptotically efficient[26]. Details of the multiple imputation are described elsewhere [3, 27].

### Contextual-level measures

In this study, we investigated each PA facility separately, as follows: number of sports courts (zero, one, two, three or more), presence of running/athletics track (yes or no), schoolyard with teacher-directed PA (no schoolyard; schoolyard not intended for teacher-directed PA; schoolyard available and intended for teacher-directed PA), availability swimming pool (not available or not in usable condition; available in usable condition) and locker room (not available or not in usable condition; available in usable condition).

We also created a variable “number of PA facilities” at the school by adding the number of different facilities (yes = 1; no = 0) (sports courts, running/athletics track, schoolyard with teacher-directed PA, swimming pool in usable condition, and locker rooms in usable condition). The variable could range from zero (no facility available) to five (all facilities evaluated were available and in usable condition). For instance, the school was classified as 5 in the number of PA facilities variable if: the number of sports courts was ≥1, a running/athletics track was available, a schoolyard was available and intended for teacher-directed PA, a swimming pool was in usable condition, and a locker room was available in usable condition. Finally, we investigated (separately) the offer of extracurricular sports activities and its costs (no activities available; paid activities only; unpaid activities only; paid and unpaid activities available).

The number of students enrolled in the school and type of school were included as contextual-level covariates.

### Statistical Analysis

We first calculated the prevalence of PE classes, LTPA, and PAL. We then used a two-level logistic regression (school and individual) model to evaluate the association between school context measures and each PA domain, whereby unadjusted and adjusted odds ratios (ORs) were calculated. Due to the hierarchical nature of the data (students nested in schools), and assuming equal correlation between students from the same school and independence for different schools, we performed multilevel logistic regression models comprising the following four steps:

1. We determined the variability of each dependent variable (PE classes, LTPA, and PAL) across schools (empty model), which indicated the school clustering. We used the 'latent variable method' to obtain intraclass correlation coefficient (ICC), assuming that PA practice follows a logistic distribution, with 3.29 individual-level variance [28]. We also verified the proportional change in variance of each dependent variable across schools using two steps: i) including sociodemographic variables, number of students enrolled in the school and type of school; ii) step 1 variables, number of PA facilities and offer of extracurricular sports activities.
2. For each dependent variable, we performed a bivariate analysis (unadjusted model) with number of PA facilities, extracurricular sports activities, and each school-level characteristic (sports courts, running/athletics track, swimming pool, schoolyard with teacher-directed PA, and locker rooms).
3. We performed multivariate analysis using several models for each dependent variable (PE classes, LTPA, and PAL). We ran two models for each outcome: firstly, one model including only number of PA facilities and, secondly, another including only extracurricular sports activities (independent variables). Both models were adjusted by the following covariates: age (in years), sex, mother’s education, region and municipality, number of students enrolled in the school and type of school.
4. We then also performed a multivariate analysis including PA facilities separately (number of sports courts, running/athletics track, swimming pool, schoolyard with teacher-directed PA, locker rooms) for each dependent variable. All multivariate analyses were adjusted by the following covariates: age (in years), sex, mother’s education, region and municipality, and type of school. For the number of sports courts model, we also adjusted by number of students enrolled in the school.

The analyses were performed using Stata 12.1 software (StataCorp LP, College Station, TX, USA), taking into account the sampling design of the survey (this was not possible for the multilevel models due to operational limitations of the software) and assuming a statistical significance if alpha-level was less than 5%.

### Ethical considerations

The PeNSE was approved by the National Commission for Research Ethics (Brazilian Ethics Committee) (record no. 16 805), according to the Declaration of Helsinki, and all participants gave informed consent through a self-administered questionnaire (using smartphones). Informed consent from the parents, careers, or guardians was not obtained on behalf of the participants because the Brazilian Statute of Children and Adolescents (Law n° 8.069; 13 July, 1990) gives adolescent autonomy to takes initiatives, such as answering questionnaires that offer no risk to health and which have the clear purpose of supporting health policies for this age group. All these consent procedures were approved by the National Commission for Research Ethics. Access to the study database is freely available through the Brazilian Institute of Geography and Statistics website with no identifying information on the participants (data are anonymized).

## Results

Almost two-thirds of the students were aged 14 to 15 years, and nearly half were male (48%) and had mothers with secondary education (Table 1).

**Table 1:** Individual and school-level characteristics in adolescents. Brazil, 2012.

Variable	Total	
	n	%
**Individual-level characteristics**		
**Sex**		
Male	52,015	48.0
Female	57,089	52.0
**Age**		
<13	22,443	22.9
14–15	72,005	63.9
>16	14,656	13.2
**Mother’s education level**		
Incomplete elementary	37,629	39.0
Complete elementary	18,978	17.8
Complete secondary	35,448	30.7
Complete higher	17,015	12.5
School-level characteristics**		
**Type of school**		
Public	2,234	79.0
Private	608	21.0
PA facilities and extracurricular sports activities in school**		
How many sports courts*		
0	612	24.3
1	1,676	60.7
2	372	10.9
3 or more	174	4.0
**Running/athletics track**		
No	2,760	98.1
Yes	74	1.7
**Schoolyard with teacher-directed PA**		
No schoolyard	109	4.1
Schoolyard not available for teacher-directed PA	1,126	43.2
Schoolyard available and intended for teacher directed PA	1,599	52.6
**Swimming pool in usable condition**		
No swimming pool/ Not in usable condition	2,546	92.1
Swimming pool available in usable condition	288	7.9
**Locker room in condition to use**		
No locker room/ Not in usable condition	971	72.5
Locker room available in usable condition	1,863	37.0
**Numberof PA facilities**		
0	263	12
1	942	34.3
2	1,016	34.5
3	439	15.3
4	160	3.7
5	14	0.2
**Extracurricular sports activities**		
None available	945	35.7
Paid activities only	212	6.3
Paid and unpaid activities	112	2.8
Unpaid activities only	1,565	55.0

* 8 missing schools (0,2%).

** n and % refer to number and proportion of schools, respectively.

Most of the schools were public, had at least one sports court, and offered extracurricular sports activities (64%). Availability of a schoolyard with teacher-directed PA, swimming pool in usable condition, and locker room in usable condition were less common. (Table 1).

The ICC estimated in the empty model demonstrated that 57% of the variance of PE classes, 2.4% of the variance of LTPA and 3.2% of the variance of PAL are at school level, indicating the multilevel structure of our dependent variables. When sociodemographic variables, number of students enrolled in the school and type of school were included in the model, the proportional change in variance was 18.9% for PE classes, 30% for LTPA and 26.8% for PAL; When the number of PA facilities and extracurricular sports activities were included in the previous model, the proportional change in variance was19.9% for PE classes, 33.1% for LTPA and 29.6% for PAL. In other words, the proportional change in variance accounting for PA facilities and extracurricular sports activities was 1% for PE classes, 3.1% for LTPA, and 3.1% for PAL.

Table 2 shows the association of number of PA facilities and extracurricular sports activities with PE classes, LTPA and PAL. For PE classes, there is a gradient, although not linear, between number of PA facilities and the odds of PE classes. However, for LTPA the association was found only in schools with at least four PA facilities. For the minimum recommended PAL, there is a less pronounced gradient, from as least two PA facilities. Regarding extracurricular sports activities: paid only and paid/unpaid activities were consistently associated with LTPA and free of charge activity was associated with PAL.

**Table 2:** Association between number of PA facilities with physical education classes, leisure-time physical activity and physical activity level in adolescents—multilevel analysis.

Variable		Physical Education Classes		Leisure-time Physical Activity		Physical Activity Level	
	Proportion of Schools	Unadjusted model	Full Model*	Unadjusted model	Full Model*	Unadjusted Model	Full Model*
	% (95% CI)	OR (95% CI)	OR (95% CI)	OR (95% CI)	OR (95% CI)	OR (95% CI)	OR (95% CI)
Number of PA facilities**	** **						
0	12.0 (7.4–16.5)	1	1	1	1	1	1
1	34.3 (26.5–42.0)	**2.64 (1.96–3.56)**	**1.59 (1.20–2.10)**	1.05 (0.98–1.12)	1.01 (0.94–1.07)	**1.19 (1.10–1.29)**	1.06 (0.98–1.15)
2	34.5 (27.8–41.3)	**3.81 (2.84–5.12)**	**1.94 (1.45–2.58)**	**1.10 (1.04–1.18)**	1.02 (0.96–1.09)	**1.37 (1.26–1.48)**	**1.15 (1.06–1.24)**
3	15.3 (11.7–19.0)	**2.50 (1.80–3.49)**	**1.67 (1.20–2.34)**	**1.12 (1.05–1.21)**	1.00 (0.93–1.08)	**1.38 (1.26–1.50)**	**1.14 (1.04–1.25)**
4	3.7 (2.8–4.6)	**3.26 (2.13–4.99)**	**2.76 (1.77–4.31)**	**1.41 (1.29–1.55)**	**1.14 (1.03–1.26)**	**1.56 (1.41–1.74)**	**1.22 (1.08–1.37)**
5	0.2 (0.0–0.4)	**10.19 (3.22–32.27)**	**7.96 (2.71–23.39)**	**1.63 (1.28–2.08)**	**1.40 (1.10–1.78)**	**1.94 (1.51–2.51)**	**1.55 (1.20–2.00)**
Extracurricular sports activities**					** **	** **	** **
None available	35.7 (30.5–41.0)	1	1	1	**1**	1	1
Paid activities only	6.3 (5.2–7.4)	1.16 (0.83–1.60)	1.32 (0.93–1.94)	**1.32 (1.23–1.41)**	**1.13 (1.04–1.23)**	**1.22 (1.13–1.33)**	0.96 (0.87–1.05)
Paid and unpaid activities	2.8 (1.8–3.8)	1.07 (0.70–1.64)	1.38 (0.89–2.14)	**1.38 (1.26–1.51)**	**1.18 (1.08–1.31)**	**1.24 (1.12–1.37)**	1.00 (0.89–1.12)
Unpaid only	55.1 (50.5–59.7)	**1.24 (1.04–1.48)**	** **1.16 (0.99–1.37)	1.03 (0.99–1.07)	1.01 (0.98–1.05)	**1.10 (1.05–1.15)**	** 1.08 (1.03–1.12)**

*adjusted by age (in years), sex, mother’s education level, region, municipality, type of school, and number of enrolled students in the school (for number of PA facilities only).

** Number of PA facilities and extracurricular sports activities models were run separately.

Table 3 shows the results of the association between school-level characteristics (PA facilities and extracurricular sports activities) and each domain of PA. Schools with one (OR 1.48; 95% CI 1.22 to 1.80), two (OR 2.37; 95% CI 1.78 to 3.17), and three or more sports courts (OR 4.43; 95% CI 3.00to 6.56) presented a higher odds of student participation in PE classes. Having a swimming pool available in usable condition was also associated with higher odds of PE classes’ participation (OR 1.47; 95% CI 1.08 to 1.94) (Table 3).

**Table 3:** Association between school level variables with physical education classes in adolescents—multilevel analysis.

PA facilities in school	Physical Education classes (≥2 week)					
	Prevalence		Unadjusted Model		Full Model*	
	%	(95% CI)	OR	(95% CI)	OR	(95% CI)
How many sports court**						
0	29.7	(24.7–34.8)	1		1	
1	37.4	(25.4–49.3)	**1.91**	**(1.57–2.33)**	**1.48**	**(1.22–1.80)**
2	50.7	(41.3–60.0)	**3.84**	**(2.92–5.05)**	**2.37**	**(1.78–3.17)**
3 or more	58.9	(46.7–71.2)	**5.98**	**(4.18–8.57)**	**4.43**	**(3.00–6.56)**
**Running/athletics track**						
No	38.6	(28.4–48.8)	1		1	
Yes	44.3	(34.7–53.9)	1.62	(0.98–2.69)	1.10	(0.69–1.75)
**Schoolyard with teacher-directed PA**						
No schoolyard	33.6	(25.7–41.5)	1		1	
Schoolyard not available for teacher-directed PA	36.6	(27.3–45.9)	1.5	(0.98–2.31)	0.93	(0.62–1.38)
Schoolyard available and intended for teacher directed PA	40.8	(28.9–52.8)	**2.21**	**(1.44–3.38)**	1.23	(0.83–1.83)
**Swimming pool**						
No swimming pool/ Not in usable condition	39.0	(29.9–48.1)	1		1	
Swimming pool available in usable condition	34.8	(11.6–58.1)	0.91	(0.70–1.19)	**1.47**	**(1.08–1.94)**
**Locker room**						
No locker room/ Not in usable condition	41.0	(34.9–47.1)	1		1	
Locker room available in usable condition	33.0	(14.2–51.8)	0.87	(0.73–1.04)	1.42	(0.88–1.28)

*Adjusted by age (in years), sex, mother’s education level, region, municipality, and type of school.

** Adjusted by age (in years), sex, mother’s education level, region, municipality, type of school, and number of enrolled students in the school.

Presence of two (OR 1.07; 95% CI 1.01 to 1.22) or three or more sports courts (OR 1.21; 95% CI 1.11 to 1.32), running/track athletics (OR 1.10; 95% 1.01 to 1.22) and swimming pool available in usable condition (OR 1.08; 95% CI 1.01–1.16) were associated with LTPA participation (Table 4).

**Table 4:** Association between school level variables with leisure-time physical activity in adolescents—multilevel analysis.

Variable	Leisure-time physical activity (≥1 week)					
	Prevalence		Unadjusted Model		Full model*	
	%	(95% CI)	OR	(95% CI)	OR	(95% CI)
How many sports court**						
0	65.7	(63.0–68.4)	1		1	
1	67.5	(67.2–67.8)	**1.06**	**(1.02–1.11)**	1.01	(0.97–1.05)
2	68	(65.8–70.1)	**1.16**	**(1.09–1.23)**	**1.07**	**(1.01–1.15)**
3 or more	73.8	(71.5–76.0)	**1.36**	**(1.27–1.47)**	**1.21**	**(1.11–1.32)**
**Running/athletics track**						
No	67.4	(66.6–68.2)	1		1	
Yes	72.8	(69.6–76.1)	**1.24**	**(1.12–1.38)**	**1.10**	**(1.01–1.22)**
**Schoolyard with teacher-directed PA**						
No schoolyard	68.7	(66.8–70.7)	1		1	
Schoolyard not available for teacher-directed PA	67.4	(66.4–68.5)	**0.97**	**(0.88–1.07)**	0.91	(0.83–1.00)
Schoolyard available and intended for teacher directed PA	67.5	(66.6–68.4)	**0.99**	**(0.90–1.09)**	0.91	(0.83–1.00)
**Swimming pool**						
No swimming pool/ Not in usable condition	67.1	(66.3–67.9)	1		1	
Swimming pool available in usable condition	73.3	(70.3–76.2)	**1.27**	**(1.20–1.34)**	**1.08**	**(1.01–1.16)**
**Locker room**						
No locker room/ Not in usable condition	67.0	(65.8–67.8)	1		1	
Locker room available in usable condition	69.2	(66.9–71.5)	**1.11**	**(1.07–1.15)**	1.04	(0.99–1.08)

*Adjusted by age (in years), sex, mother’s education level, region, municipality, and type of school.

** Adjusted by age (in years), sex, mother’s education level, region, municipality, type of school, and number of enrolled students in the school.

The achievement of a minimum recommended PAL (>420 min/week) was associated (adjusted model) with the following PA facilities: sports courts (three or more = OR 1.18; 95% CI 1.07 to 1.31), schoolyard with teacher-directed PA (OR 1.15; 95% CI 1.02 to 1.29), and swimming pool available in usable condition (OR 1.10; 95% CI 1.02 to 1.18) (Table 5).

**Table 5:** Association between school level variables with physical activity level in adolescents—multilevel analysis.

Variable	Physical Activity Level (≥420 min/week)					
	Prevalence		Unadjusted Model		Full model*	
	%	(95% CI)	OR	(95% CI)	OR	(95% CI)
How many sports courts**						
0	14.6	(13.1–16.2)	1		1	
1	18.5	(17.7–19.2)	**1.26**	**(1.19–1.32)**	**1.13**	**(1.07–1.20)**
2	20	(19.1–20.9)	**1.42**	**(1.32–1.52)**	**1.19**	**(1.10–1.28)**
3 or more	23.4	(22.1–24.6)	**1.49**	**(1.37–1.62)**	**1.19**	**1.08–1.31)**
**Running/athletics track**						
No	18.1	(17.5–18.7)	1		1	
Yes	22.3	(19.5–25.1)	**1.25**	**(1.11–1.40)**	1.09	(0.97–1.22)
**Schoolyard with teacher-directed PA**						
No schoolyard	14.1	(12.5–15.6)	1		1	
Schoolyard not available for teacher-directed PA	17.9	(17.2–18.5)	**1.30**	**(1.16–1.46)**	1.13	(1.00–1.27)
Schoolyard is available and intended for teacher directed PA	18.6	(18.0–19.3)	**1.37**	**(1.22–1.54)**	**1.15**	**(1.02–1.29)**
**Swimming pool**						
No swimming pool/ Not in usable condition	17.9	(17.2–18.6)	1		1	
Swimming pool available in usable condition	21.6	(18.7–24.4)	**1.22**	**(1.14–1.30)**	**1.10**	**(1.02–1.18)**
**Locker room**						
No locker room/ Not in usable condition	17.3	(16.4–18.0)	1		1	
Locker room available in usable condition	20.3	(18.7–21.8)	**1.12**	**(1.07–1.17)**	1.03	(0.98–1.08)

*Adjusted by age (in years), sex, mother’s education level, region, municipality, and type of school.

** Adjusted by age (in years), sex, mother’s education level, region, municipality, type of school, and number of enrolled students in the school.

## Discussion

Our study is the first to use nationally representative data from an upper-middle-income country population (Brazilian adolescents) to show an association between PA facilities availability and the offer of extracurricular sports activities in schools and participation in PE classes, engagement in LTPA, and PAL. Until now, most studies on adolescent health in Brazil have focused only on individual determinants, generally exploring a single domain of PA. Our results indicate that PA facility associations differed by PA domain, adding evidence to studies on school environment and physical activity in adolescents.

It is worth mentioning that the variance accounted for by school-level was higher for PE classes (57%) than for LTPA (2.4%) and for PAL (3.2%). In fact, this difference in the magnitude of the relationship was expected since the school environment tends to have more influence on PA at school, than that performed outside school. This is particularly true in the Brazilian context, where community access to school grounds is rare. We also verified that including the number of PA facilities and extracurricular sports activities in the model explained part of the variance accounted for by school-level in all PA measures. Nevertheless, part of the school-level variance remained unexplained, which suggests that other factors, not explored in this study, may contribute to the variability in PA between schools.

The potential influence of a favorable school context on PA has been a matter of discussion in recent years [10–12]. Studies conducted in Ontario (Canada)[10], Minnesota, United States of America (US)[11] and San Diego (US)[12] consistently observed the influence of school context on MVPA at school for students between 6^th^ and 12^th^ grade, to be similar to that identified in our study. These cases showed, differences in school level accounted for 3.0% of the variance in MVPA at school (grade 9–12) in Ontario[10], 25% of the variance in total MVPA participation among boys and 15% among girls (aged 10-17-years-old) in Minnesota[11], and 42% of the variance in MVPA at school among girls and 59% among boys (grades 6–8) in San Diego, US[12]. However, none of these studies explored such a wide variety of outcomes, as our study. The differences between studies may be explained in part by sampling sociocultural differences (i.e. shared-use patterns of student and community recreational resources), variations in the measure of PA, use of different cutoffs, and the inclusion of different variables in the full model. For example, two studies only assessed variables of school environment[10, 12], while another also explored social correlates[11], potentially explaining the differences found in the magnitude of the results. Similarly, our approach, which was multilevel, involving both school context and individual aspects–provides important insights for discussion to the extent that it can reflect the relationship of interest in a more accurate way.

In this study we found an association between the number of PA facilities, the number of sports courts and the availability of a swimming pool in usable condition, and participation in PE classes. Similarly, other studies have shown that students spent more time in PE classes in schools that had sports court availability[29] and schools that offered daily PE classes or provided a separate room for PA[10].A previous study also concluded that students attending schools with the lowest number of physical environment features may benefit from increased MVPA if there is investment to improve the physical environment at the school[30]. Therefore, improving the amount, variety and condition of the facilities in the school environment may be relevant strategies to increase PA at school and enhance the participation in PE classes.

Our study also showed that school environment is important for promoting LTPA, but for this PA domain the number of PA facilities was more important than provision of PE classes. This is possibly due to the freedom of choice of LTPA, allowing diversity and the opportunity to do activities alone or in groups, unlike PE classes in which activities are mainly organized for groups, following curriculum guidelines. These findings support previous studies that found that a number of PA facilities increased the probability of students participating in unorganized PA and playing sports[31]. Qualitative studies have also identified lack of space and PA facilities among the main barriers to PA during recess among Danish students[32]. Finally, in Belgium, participation in extracurricular activities in adolescents (mean age 13 years) was positively related to the number of activities offered and provision of supervision [33]. These findings suggest that school context also plays an important role in LTPA.

Moreover, in order to adhere to the PA guidelines, schools must increase the availability of PA facilities, enabling students to extend their activities beyond PE classes, and increase LTPA through participation in extracurricular sports activities. Similarly, Sallis et al.[12]showed that schools with improved PA facilities and supervised activities are more likely to stimulate students to be physically active. These are important results because several health benefits related to PA (e.g. adiposity level, cardiometabolic health, mental health, academic self-concept, bone strength, and physical fitness) could be achieved when school-aged children and youths participate in 60 or more minutes of MVPA [1, 2]. However, it is important to highlight that even modest amounts of PA (e.g. 30 minutes/day) can have health benefits, especially among high-risk youngsters (e.g. those who are obese)[1]. Therefore, rather than meet the current PA guidelines, the main purpose of the availability of PA facilities and the offers of extracurricular sports activities should be to promote incremental increases in daily PA among all students.

Our study has several practical implications. A wide number of PA facilities must be implemented in schools across our country, as this might help to offer a diversity of opportunities in PE classes and LTPA (extracurricular sports activities). In PE classes, schools would enhance the achievement of goals established in the National Curriculum Parameters by providing proper environments in which to teach content such as sports, games, combat, gymnastics, and rhythmic and expressive activities[23]. For LTPA, offering a diversity of extracurricular sports activities may increase the probability of the students, including those less active in this domain, becoming engaged in the activities promoted by the school. Furthermore, the school could be used as a meeting point for students and the local community, where education and health issues could be discussed and practiced. This proposal for a diverse infrastructure and open school would enhance the capability of the school to promote societal knowledge related to body culture and understanding of concepts such as leisure goals, expression of feelings, affections and emotions, and opportunities for promotion, restoration and maintenance of health, one of the aims of the *Programa Saúde na Escola* (Health in Schools Program), in Brazil[34].

Although we found a relationship between PA facilities and PA practice, it is important to emphasize that the existence of the features is a proxy for the organization of school activities. School policies which encourage investment in PA facilities in the school are needed. Physical resources contribute decisively, but they alone do not promote PA, if the whole school and policymakers are not committed to it. Thus, educational and health policy makers should consider that a diverse, attractive and enjoyable school environment in terms of PA facilities and extracurricular sports activity programs might be a priority if we want to promote this behavior. In the future, different methodological approaches, such as qualitative studies, could provide knowledge about organizational flows at school, teacher-education policy, curriculum structure and other important aspects that influence how adolescents relate to PA practices.

Our study has some limitations. Initially, PA and covariates were collected through self-report questionnaires. However, a previous validation study showed high accuracy (73.1% to 92.4%) for the PA questions used in the PeNSE[35]. Furthermore, a previous study also found that students in the 9^th^year of elementary school were able to understand the questions involving the selection of categories and left few questions blank [21]. These results justify the use of self-report questionnaires in large representative surveys, such as the PeNSE. Second, regarding the number of PA facilities, we assumed the same importance for PA practice among all five PA facilities selected. From a system approach, we understand that there is a complex interaction among PA correlates[36], which might not be captured using summary scores, despite providing information regarding the variety of the context.

To conclude, schools with a higher number of PA facilities had a higher participation in PE classes and LTPA among students, and consequently were more likely to meet the PA guidelines. Extracurricular sports activities in schools were positively associated with LTPA and PAL. The number of sports courts in a school was the PA facility most strongly associated with PE classes, LTPA, and PAL. In summary, school context has important potential to increase the engagement of adolescents in PA. Therefore, school environment has a fundamental role in allowing adolescents, who spend part of their day in school, to have a more active lifestyle.
